# Competing endogenous RNA networks in ovarian cancer: from bench to bedside

**DOI:** 10.17179/excli2024-7827

**Published:** 2025-01-07

**Authors:** Roghaiyeh Derogar, Fatemeh Nejadi Orang, Mahdi Abdoli Shadbad

**Affiliations:** 1 Fellowship in Gynecologic Oncology, Department of Gynecology, Faculty of Medical Sciences, Tabriz Medical Sciences, Islamic Azad University, Tabriz, Iran; 2 Immunology Research Center, Tabriz University of Medical Sciences, Tabriz, Iran; 3 Department of Immunology, Faculty of Medicine, Tabriz University of Medical Sciences, Tabriz, Iran

**Keywords:** circular RNA, competing endogenous RNAs, long non-coding RNA, microRNAs, ovarian cancer

## Abstract

Epithelial ovarian cancer is responsible for the majority of ovarian malignancies, and its highly invasive nature and chemoresistant development have been major obstacles to treating patients with mainstream treatments. In recent decades, the significance of microRNAs (miRNAs), circular RNAs (circRNAs), long non-coding RNAs (lncRNAs), and competing endogenous RNAs (ceRNAs) has been highlighted in ovarian cancer development. This hidden language between these RNAs has led to the discovery of enormous regulatory networks in ovarian cancer cells that substantially affect gene expression. Aside from providing ample opportunities for targeted therapies, circRNA- and lncRNA-mediated ceRNA network components provide invaluable biomarkers. The current study provides a comprehensive and up-to-date review of the recent findings on the significance of these ceRNA networks in the hallmarks of ovarian cancer oncogenesis, treatment, diagnosis, and prognosis. Also, it provides the authorship with future perspectives in the era of single-cell RNA sequencing and personalized medicine.

## Introduction

Ovarian cancer is one of the most worrisome gynecological cancers that carries a poor prognosis. In the United States, it has been estimated that 19,680 cases are diagnosed with ovarian cancer, and ovarian cancer is responsible for 12,740 deaths in 2024. In the United States, ovarian cancer is the fifth leading cause of death by sex in 2024 (Siegel et al., 2024[116]). From the histopathological point of view, ovarian cancer is classified into four major groups, i.e., serous, endometrioid, clear cell, and mucinous neoplasms (Arora et al., 2021[5]). Epithelial ovarian cancer stems from the epithelium of the ovarian surface and is responsible for more than 85 % of cases (Atallah et al., 2023[7]). The mainstream treatment for ovarian cancer patients is surgery and chemotherapy, including platinum-based agents; however, patients can experience tumor relapse (Tewari et al., 2019[126]). Besides, most ovarian cancer cases are diagnosed in advanced stages, and the five-year survival of advanced ovarian cancer patients is dismal (Torre et al., 2018[127]). Therefore, there is an urgent need to develop novel approaches for early diagnosis and treatments for ovarian cancer patients. 

Based on Weinberg et al., sustained proliferation, resisting cell death, replicative immortality, angiogenesis, invasion, sustained growth, and metastasis, along with metabolism reprogramming and immune system evasion, are the hallmarks of cancer (Hanahan and Weinberg, 2011[49]). Increased expression of oncogenes and tumor suppressor genes' downregulation contribute to oncogenesis and the development of cancer hallmarks (Shadbad et al., 2022[112]). Non-coding RNAs (ncRNAs) constitute approximately 98 % of the human genome; ribosomal RNA, responsible for protein synthesis, transfer RNA, responsible for bringing amino acids, small nucleolar RNA, responsible for mRNA maturation and post-transcriptional modifications, endogenous small-interfering RNA, involved in gene regulation, PIWI-interacting RNA, involved in gene regulation, long non-coding RNAs (lncRNAs), and microRNA (miRNA) are among the ncRNAs that have vital roles in gene expression (Le et al., 2021[62]). MiRNAs are approximately 22 nucleotides-long ncRNAs that post-transcriptionally silence target mRNAs (O'Brien et al., 2018[94]). Primary miRNAs result from the RNA polymerase II-mediated transcription of miRNA genes in the nucleus. Following microprocessor-mediated primary miRNA processing, precursor miRNAs are produced. Then, the further processing of precursor miRNAs by DICER1, as a ribonuclease III enzyme, produces mature miRNAs. The loading of the guide strand of mature miRNAs to the miRNA-induced silencing complex leads to degradation and translational repression of target mRNA in a complementary binding manner (Lin and Gregory, 2015[69]). LncRNAs contain more than 200 nucleotides that are generally transcribed by RNA polymerase II. Based on the function, lncRNAs are classified into five types, i.e., signaling (molecular signals in response to various stimuli), decoy (binding to mi-RNAs to liberate target mRNA), scaffold (forming ribonucleoprotein complexes with proteins to regulate chromosome rearrangement, histone modification, and RNA polymerase II activity), guide (forming ribonucleoprotein complexes for regulating transcription of target genes), and SINEUPs (increasing the translation of genes) (Liu et al., 2021[79]). Circular RNAs (circRNAs) are newly discovered RNAs that can be divided into three categories based on their origin, i.e., exonic circRNAs, exon-intron circRNAs, and circular intronic RNAs. miRNA sponge, modulating RNA stability, and interacting with RNA-binding proteins are the identified functions of circRNAs (Ma et al., 2020[87]). In this regard, Salmena et al. have highlighted the cross-talk between these ncRNAs and identified a "language," i.e., microRNA response elements (MREs), between lncRNA with miRNAs, leading to the regulation of mRNA expression (Salmena et al., 2011[110]). CircRNAs and lncRNAs possess MREs that establish dynamic competition with mRNAs for binding to miRNAs. This idea justifies the presence of circRNA/miRNA/mRNA and lncRNA/miRNA/mRNA axes that are dysregulated in various cancers (Yang et al., 2016[146]). Mounting evidence has highlighted the importance of circRNA- and lncRNA-mediated ceRNA networks in governing various aspects of oncogenesis in different cancers, like glioblastoma (Zhu et al., 2020[161]), breast adenocarcinoma (Fiscon et al., 2023[35]), lung adenocarcinoma (Wang et al., 2021[135]), colorectal carcinoma (Wang et al., 2019[131]), and pancreatic cancer (Xu et al., 2022[144]). Because circRNA- and lncRNA-mediated ceRNA networks regulate an extensive number of genes and pathways, identifying oncogenic and tumor-suppressive ones can provide invaluable insights into specific cancer treatment, diagnosis, and prognosis of affected patients. The current study aimed to provide the authorship with a comprehensive and up-to-date review of the well-established pathways in different aspects of ovarian cancer oncogenesis and the related ceRNA networks; this information can be considered a blueprint for future promising targets for diagnosis, prognosis, and treatment of ovarian cancer patients. 

### Search strategy

The present study reviews the recent advances in the significance of ceRNAs on the hallmarks of ovarian cancer, i.e., proliferation, cell cycle, migration, invasion, angiogenesis, stemness, metabolic reprogramming, apoptosis, ferroptosis, and pyroptosis, using the Scopus, Embase, PubMed, and Web of Science databases. Besides studying their value as novel biomarkers, their significance in ovarian cancer chemotherapy, immunotherapy, and epigenetics was reviewed. In each heading, the well-established pathways involved in the abovementioned oncogenesis hallmarks are briefly discussed, and then the related ceRNA networks and their components will be reviewed. 

## ceRNA Networks in the Proliferation and Cell Cycle of Ovarian Cancer Cells

Tumor cell proliferation is one of the hallmarks of cancers (Hanahan and Weinberg, 2011[49]). The PI3K/Akt/mTOR oncogenic signaling pathway is implicated in epithelial ovarian cancer development (López-Reig and López-Guerrero, 2020[81]). As lipid kinases, PI3Ks are the downstream targets of the G protein-coupled receptors and receptor tyrosine kinases; interaction of ligands with specific receptor tyrosine kinases leads to their phosphorylation of intracellular domains and adaptor proteins, resulting in the activation of PI3K (Gasparri et al., 2017[41]). Activated PI3K generated PIP3 from PIP2, paving the way for the phosphorylation of AKT kinase and AKT activation. PTEN has been identified as a crucial regulator of this pathway via converting PIP3 to PIP2; besides, INPP4B can generate PIP from PIP2 via the dephosphorylation of PIP2. Activated AKT can directly and indirectly activate mTORC1, leading to cell proliferation (Ediriweera et al., 2019[30]). The following discusses the current evidence of the significance of ceRNA networks in the proliferation and PI3K/Akt/mTOR of ovarian cancer cells (Table 1(Tab. 1); References in Table 1: Dong et al., 2021[27]; Gong et al., 2020[44]; Guo et al., 2020[47]; Li et al., 2020[66]; Wang et al., 2021[133], 2022[132]).

It has been reported that miR-484-5p is decreased in ovarian cancer tissues and cell lines, and its ectopic expression decreases cell proliferation *in vitro* and *in vivo* via targeting the SRC/p-PI3K/p-AKT pathway (Yang et al., 2020[147]). Cui et al. have shown that suppressing miR-484-5p increases the proliferation of ovarian cancer cells (Cui and Li, 2021[22]). Wang et al. have highlighted the significance of SNHG17/miR-485-5p/AKT1 in ovarian cancer cells. As a pro-tumoral lncRNA, SNHG17 targets miR-484-5p expression, increasing AKT1 and p-mTOR expression in ovarian cancer cells (Wang et al., 2022[132]). Dong et al. have reported that the expression levels of lncRNA GAS5 and PTEN are downregulated in ovarian cancer tissues and cell lines; however, the expression of miR-96-5p is upregulated; GAS5 overexpression sponges miR-96-5p expression, leading to regulation of PI3K/Akt/mTOR and decreased proliferation of ovarian cancer cells (Dong et al., 2021[27]). In line with this, miR-96-5p expression is upregulated in serum and tumoral tissues of ovarian cancer patients, and miR-96-5p increases the proliferation of ovarian cancer cells via increasing the AKT phosphorylation and cyclin D1 expression (Liu et al., 2019[72]). In ovarian cancer cells, the expression levels of miR-21 and PTEN are upregulated and downregulated, respectively. Also, the ectopic expression of miR-21 inhibitor increases PTEN expression, leading to the decreased proliferation of ovarian cancer cells (Liu et al., 2019[74]). As a downregulated circRNA in ovarian cancer tissues and cell lines, circRNA-9119 sponges miR-21-5p, leading to decreased ovarian cancer cell proliferation via the PTEN-Akt pathway (Gong et al., 2020[44]). As a pro-tumoral lncRNA, MALAT1 increased expression upregulates the expression of p-PI3K and p-AKT, leading to tumor proliferation and ovarian cancer development (Mao et al., 2021[89]). 

**Figure 1:**
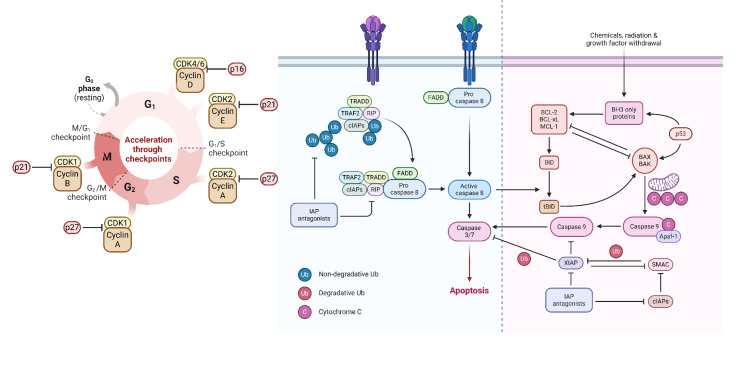
The cell cycle and apoptosis pathways A) The cell cycle consists of G_1_, S, G_2_, and M phases that are regulated by proto-oncogenes and tumor-suppressive genes. B) Intrinsic and extrinsic apoptosis pathways are two distinct pathways that mediate apoptosis by converging into caspase 3 and caspase 7.

Dysregulated cell cycle is one of the characteristics of cancer cells. The interactions between cyclins, cyclin-dependent kinases (CDKs), and cyclin-dependent kinase inhibitors regulate the mitotic cell cycle. In quiescence, the inactivation of CDK4/6 and low expression levels of cyclins suppress the cell cycle. Meanwhile, Rb inhibits the activation of E2F transcription factors, leading to inhibited expression of pro-proliferative factors (Dall'Acqua et al., 2021[25]). Under mitogenic stimuli, cyclin D with CDK4/6 phosphorylates Rb, leading to Rb phosphorylation and E2F liberation. Besides, phosphorylated Rb increases the expression of cyclin E, cyclin A2, and cyclin B1. During the late G1 phase, cyclins E1 and E2 bind and activate CDK2, which is inactivated by p27^KIP1^ and p21^CIP1^; the active CDK2 can mediate the phosphorylation of p27^KIP1^ and Rb, DNA replication, histone synthesis, and centrosome duplication. The cyclin E/CDK2 active complex leads to the S phase initiation. The cyclin E is removed from the cyclin E/CDK2, and cyclin A/CDK2 is formed near the end of the S phase. The cyclin A/CDK2 complex progresses the cell cycle from the S phase to the G2 phase. Finally, the cyclin A/CDK1 complex results in the transition to the M phase (Ding et al., 2020[26]) (Figure 1A(Fig. 1)). Increased protein expression of cyclin A1 has been substantially associated with the overall survival of ovarian cancer patients, and silencing cyclin A1 improves the chemosensitivity of ovarian cancer cells to paclitaxel (Huang et al., 2016[54]). In this regard, CDK6 expression is substantially upregulated in ovarian cancer tissues compared to non-tumoral tissues, and its knockdown is associated with enhanced chemosensitivity of ovarian cancer cells to cisplatin (Duan et al., 2020[29]). Silencing cyclin D1 arrests the cell cycle in the G_0_/G_1_ phase and sensitizes ovarian cancer cells to olaparib both *in vitro* and *in vivo* (Zhong et al., 2019[156]). Increased expression of circ-0009910 in ovarian cancer tissues is associated with the advanced International Federation of Gynecology and Obstetrics (FIGO) stage, lymph node metastasis, and poor overall survival of affected patients. It has been reported that circ-0009910 ectopic expression sponges miR-145 expression, upregulates cyclin D, CDK4, and CDK6, and enhances the proliferation of ovarian cancer cells (Li et al., 2020[66]). Guo et al. have highlighted the significance of the circ-0000714/miR-370-3p axis in the cell cycle of ovarian cancer cells. Circ-0000714 knockdown or miR-370-3p ectopic expression arrests the cell cycle, increases the proportion of ovarian cancer cells in the G_1 _phase, and downregulates CDK6, CDK4, and cyclin D1 (Guo et al., 2020[47]). In this regard, Wang et al. have shed light on the significance of SNHG15/miR-370-3p/CDK6 in ovarian cancer cells. SNHG15 increased expression is associated with poor progression-free survival of ovarian cancer patients, and its knockdown arrests the cell cycle (Wang et al., 2021[133]). Ma et al. have studied the circ_0025033/has-miR-370-3p axis in ovarian cancer cells. The expression of circ_0025033 is upregulated in ovarian cancer tissues, and its upregulation is associated with poor overall survival of affected patients. Also, miR-370-3p expression is downregulated in ovarian cancer tissues, and there is a negative correlation between miR-370-3p and circ_0025033 expression in ovarian cancer. The knockdown of circ_0025033 has considerably decreased the proliferation of ovarian cancer via the circ_0025033/has-miR-370-3p/SLC1A5 axis (Ma et al., 2022[86]). The let-7 family is involved in various cancer development. Biamonte et al. have shown that let-7g is downregulated in ovarian cancer tissues compared to non-tumoral tissues, and let-7g serum level is decreased in chemoresistant patients compared to chemosensitive ones; let-7g ectopic expression considerably downregulates cyclin D2 expression, arrests the cell cycle, and enhances the chemosensitivity of ovarian cancer cells to cisplatin (Biamonte et al., 2019[10]). It has been reported that let-7d-5p is downregulated in ovarian cancer tissues and cell lines compared to non-tumoral tissues and cell lines; let-7d-5p downregulates CDK2 expression, arrests the cell cycle at the G_0_/G_1_ phase, and improves the chemosensitivity of ovarian cancer cells to cisplatin (Chen et al., 2019[18]).

## ceRNA Networks in the Regulated Cell Death of Ovarian Cancer Cells

Cell death is intertwined with life, and it can be categorized into regulated cell death and accidental cell death. Unlike accidental cell death, regulated cell death has tightly controlled signaling pathways and molecular mechanisms. Ferroptosis, pyroptosis, apoptosis, necroptosis, cuproptosis, parthanatos, lysozincrosis, disulfidptosis, entosis, NETosis, alkaliptosis, and oxeiptosis are among the regulated cell deaths (Hadian and Stockwell, 2023[48]; Peng et al., 2022[99]). The following briefly discusses the molecular mechanisms of apoptosis, ferroptosis, and pyroptosis, i.e., the regulated cell deaths that ceRNA networks have been studied on and the identified ceRNA networks in ovarian cancer (Table 2(Tab. 2); References in Table 2: Cai et al., 2022[12]; Li et al., 2021[64]; Liu et al., 2023[73]; Qin et al., 2023[103]; Tan et al., 2021[123]; Wang et al., 2020;[129] Zhu and Mei, 2021[159]; Zhu et al., 2020[160]).

Apoptosis is a form of regulated cell death that is mediated by caspases through intrinsic and extrinsic signaling pathways; DNA damage and hypoxia are responsible for the initiation of the intrinsic signaling pathway. Extrinsic cell death singlings, like Fas-L and TNF-α, are responsible for the initiation of the extrinsic signaling pathway (Wong, 2011[138]). In the intrinsic pathway, the apoptosis stimuli lead to the insertion of BAX and BAK at the mitochondrial outer membrane, resulting in the permeabilization of the mitochondrial membrane and the release of cytochrome c into the cytosol. In this regard, BCL-2 and BCL-xL prevent the release of cytochrome c and serve as anti-apoptotic factors. Following the release of cytochrome c and its binding to Apaf-1 and pro-caspase-9, the apoptosome is developed, where the pro-caspase-9 is converted to caspase-9. Afterward, caspase-3 and caspase-7 are activated, and apoptosis is initiated (Jan and Chaudhry, 2019[57]). In the extrinsic apoptosis pathway, the initiator procaspase-8 and procaspase-10 bind to the adaptor proteins like FADD and TRADD at the death receptors, developing a death-inducing signaling complex; the death-inducing signaling complex activates the procaspase-8 and procaspase-10, resulting in activation of caspases-3, caspases-6, and caspases-7, and apoptosis initiation (Pfeffer and Singh, 2018[100]) (Figure 1B(Fig. 1)). Studies have investigated apoptosis-regulating ceRNA networks in ovarian cancer fate. As an upregulated lncRNA in ovarian cancer tissues and cell lines, the increased expression of MIR4435-2HG is associated with poor overall survival and lymph node metastasis in ovarian cancer patients. MIR4435-2HG knockdown increases the apoptosis rate, downregulates BCL-2 expression of ovarian cancer cells, and decreases tumor size in animal models via the MIR4435-2HG/miR-128-3p axis (Zhu et al., 2020[160]). HAGLROS is an upregulated lncRNA in ovarian cancer cells; HAGLROS knockdown increases apoptosis rate, upregulates BAX protein expression, and downregulates BCL-2 protein expression via the HAGLROS/miR-26b-5p axis (Zhu and Mei, 2021[159]). CCAT1 is an upregulated lncRNA in cisplatin-resistant ovarian cancer cells; CCAT1 knockdown stimulates apoptosis, downregulates BCL-2, and upregulates BAX in ovarian cancer cells via the CCAT1/miR-454 axis (Wang et al., 2020[129]). Liu et al. have demonstrated that SNHG4 is an upregulated lncRNA in ovarian cancer tissues. SNHG4 knockdown is associated with increased apoptosis rate, decreased BCL-2, and increased BAX protein expression in ovarian cancer cells; these anti-tumoral effects are reversed following co-inhibition of SNHG4 and miR-98-5p (Liu et al., 2023[73]). HAND2-AS1 is a downregulated lncRNA in ovarian cancer tissues and cisplatin-resistant ovarian cancer cells, and its ectopic expression stimulates apoptosis, increases BAX protein expression, and decreases BCL-2 protein expression via regulating miR-106a-5p expression in cisplatin-resistant ovarian cancer cells (Li et al., 2021[64]). 

Ferroptosis is a recently identified iron-dependent cell death associated with reactive oxygen species and lipid peroxidation (Yan et al., 2021[145]). The beneficial effects of ferroptosis on cancer treatment have been recently discovered (Feng et al., 2023[34]). GPX4, Nrf2, ACSL4, and SLC7A11 are the regulatory genes of this regulated cell death. The cysteine/GSH/GPX4 signaling pathway, FSP1/ DHODH/CoQ10, and GCH1/BH4/DHFR axes regulate ferroptosis (Tang et al., 2021[124]). Aside from pharmacological stimulation of ferroptosis, gene therapy and leveraging the ceRNA network concepts can activate this cell death pathway and cause tumor growth inhibition (Nejadi Orang and Abdoli Shadbad, 2024[92]). The study on the significance of ceRNA networks in regulating ferroptosis in ovarian cancer is in its infancy. Cai et al. highlighted the significance of ADAMTS9-AS1/miR-587/SLC7A11 in ovarian cancer development. As an upregulated lncRNA in ovarian cancer cells, ADAMTS9-AS1 knockdown decreases the migration and proliferation of ovarian cancer cells, increases Fe^2+^, iron, and reactive oxygen species (ROS) levels, and decreases the expression levels of GPX4 and SLC7A11 via the ADAMTS9-AS1/miR-587/SLC7A11 (Cai et al., 2022[12]). Qin et al. have shed light on the significance of the circSnx12/miR-194-5p/SLC7A11 axis on ferroptosis and ovarian cancer development. The expression level of circSnx12 is upregulated in cisplatin-resistant ovarian cancer tissues compared to cisplatin-sensitive ones; circSnx12 knockdown increases the chemosensitivity of ovarian cancer to cisplatin both *in vitro* and *in vivo*, inhibits cell proliferation, stimulates apoptosis, decreases GSH, increases lipid peroxidation, and activates ferroptosis in ovarian cancer via the circSnx12/miR-194-5p/SLC7A11 (Qin et al., 2023[103]). 

Pyroptosis is another regulated cell death associated with gasdermin-mediated cell expansion and cytomembrane ruptures (Wei et al., 2022[136]). Specific pathogen- and damage-associated molecular patterns can activate the NLRP1, NLRC4, AIM2, and NLRP3, which subsequently lead to the formation of ASC focus. The ASC focus recruits procaspase-1, leading to the autocleavage and activation of caspase-1. The caspase-1-mediated activation of pyroptosis constitutes the canonical inflammasome pathway. Caspase-1 activation facilitates the secretion of IL-18 and IL-1β and gasdermin D-mediated pore formation in the plasmatic membrane. The caspase-4/5-mediated activation of pyroptosis gives rise to the non-canonical inflammasome pathway; the lipopolysaccharide or hot-derived oxidized phospholipids can activate caspase-11, leading to caspase-1 activation and pyroptosis. In a recently identified pathway, mature caspase-3 leads to gasdermin E cleavage, pore formation, and pyroptosis (Fang et al., 2020[33]). Regarding the effect of pyroptosis on oncogenesis, the recent review paper by Lu et al. concluded that even though pyroptosis induction can lead to tumoricidal effects and chemotherapeutic agents and cytotoxic T-cells can lead to anti-tumoral effects via pyroptosis, further studies are needed to elucidate pyroptosis role in anti-tumoral immune responses (Lu et al., 2021[83]). Also, the research on the effect of ceRNA-mediated pyroptosis regulation in ovarian cancer is still in its infancy. Tan et al. have studied the HOTTIP/miR-148a-3p/AKT2 axis in pyroptosis and the development of ovarian cancer. As an upregulated lncRNA in ovarian cancer tissues and cell lines, HOTTIP silencing leads to NLRP1 inflammasome-mediated pyroptosis, decreases cell viability, increases the expression of pro-pyroptosis factors, like IL-18, IL-1β, and NLRP1, and activates caspase-1 in ovarian cancer cells. However, the combined therapy with HOTTIP knockdown with miR-148a-3p inhibition reverses the pro-pyroptosis effects (Tan et al., 2021[123]). However, further studies are needed to investigate the significance of ceRNA-mediated pyroptosis regulation in ovarian cancer. 

## ceRNA Networks in the Migration and Invasion of Ovarian Cancer Cells

**Figure 2:**
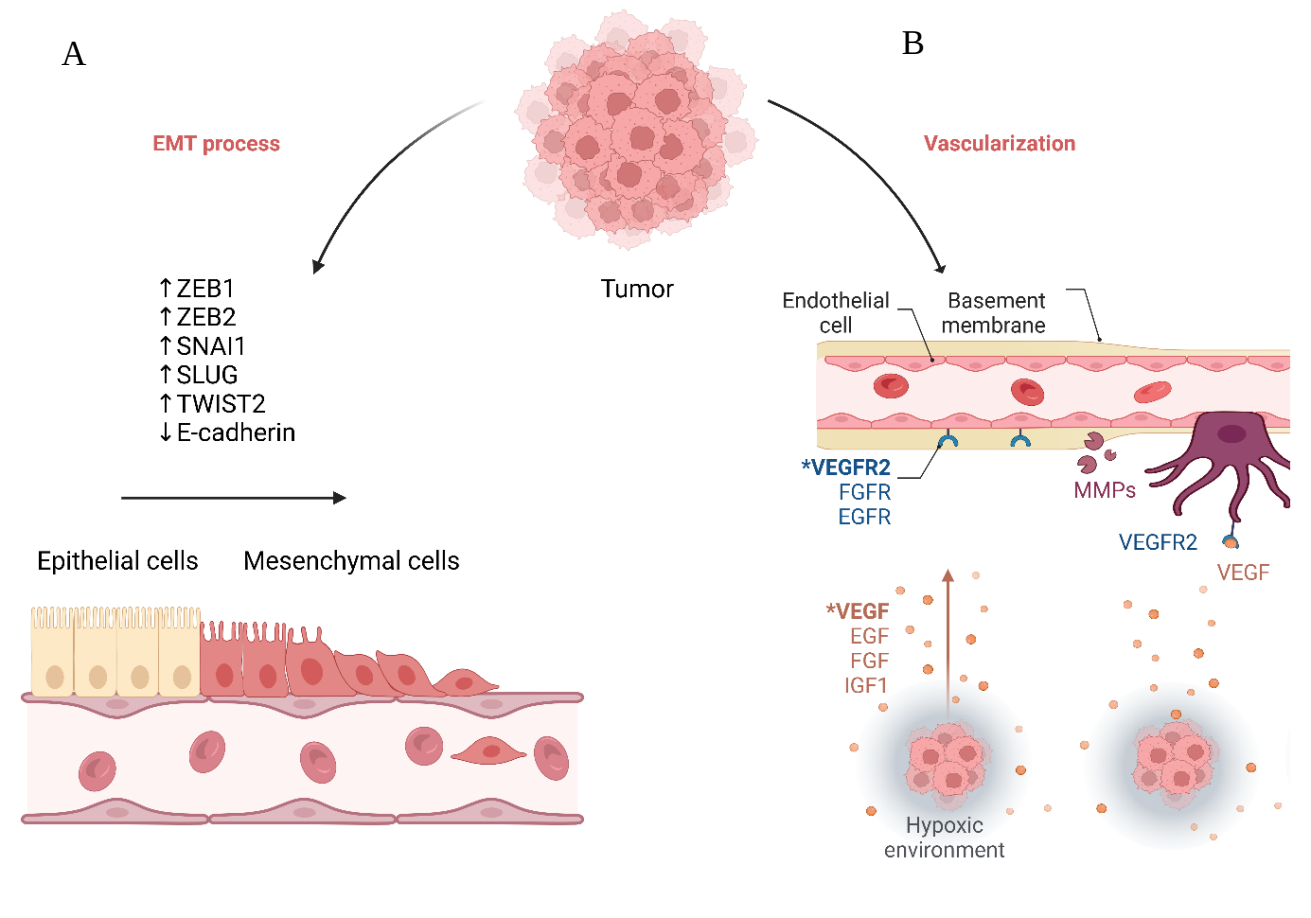
The epithelial-to-mesenchymal transition and angiogenesis in cancer. A) In the epithelial-to-mesenchymal transition, the malignant cells acquire a mesenchymal phenotype, allowing them to become more invasive. B) The tumor-released growth factors, like VEGFs, facilitate angiogenesis, which is a hallmark of malignancy.

The type III epithelial-mesenchymal transition (EMT) process is implicated in malignant tumor migration and invasion. In the EMT process, epithelial cells lose their polarity and cellular adhesion and acquire a more mesenchymal phenotype, leading to increased migration (Ribatti et al., 2020[105]). During this process, the expression of E-cadherin, as a pivotal factor in cellular junctions, is downregulated, and the expression of vimentin, fibronectin, and matrix metalloproteinases (MMPs) is increased. Various transcriptional factors, e.g., ZEB1, ZEB2, SNAI1, SLUG, and TWIST1, can regulate this process (Loret et al., 2019[82]) (Figure 2(Fig. 2)). ZEB1 belongs to the ZEB transcription factor family, characterized by 2 zinc finger clusters, mediating its DNA binding (Zhang et al., 2015[150]). It has been reported that the expression of ZEB1 is inversely associated with E-cadherin expression in ovarian cancer (Rosso et al., 2017[107]). Silencing ZEB1 upregulates E-cadherin, suppresses the migration and invasion of ovarian cancer cells *in vivo* and *in vitro*, and increases the expression of miR-200c, leading to EMT inhibition (Chen et al., 2013[15]). Consistent with this, upregulated ZEB1 expression is associated with poor overall survival of patients with high-grade serous ovarian cancer (Rae et al., 2022[104]). Li et al. have shown that the expression of ZEB2 and vimentin is increased, while E-cadherin expression is decreased in ascitic tumor cells compared to primary high-grade serous ovarian cancers; increased ZEB2 expression in primary and peritoneal metastatic high-grade serous ovarian cancer is associated with inferior overall survival and progression-free survival of affected patients. Also, the ZEB2 expression level is increased in CD133^+^ cancer stem-like cells of ovarian cancer cells, and its silencing is associated with downregulated MMP-9 expression and decreased migration (Li et al., 2021[65]). SNAIL also inhibits the transcription of E-cadherin and confers invasive properties to the epithelial cells (Cano et al., 2000[13]). Hojo et al. have shown that SNAIL knockdown suppresses the migration of ovarian cancer cells and inhibits metastasis in animal models (Hojo et al., 2018[50]). The following discusses the identified ceRNA networks in ovarian cancer migration and invasion (Table 3(Tab. 3); References in Table 3: Cao et al., 2017[14]; Liang et al., 2018[67]; Lv et al., 2022[84]; Wu et al., 2021[140]; Xiong et al., 2023[142]; Yong et al., 2018[148]).

The miR-200 family members, i.e., miR-429, miR-141, miR-200a, miR-200b, and miR-200c, substantially regulate the EMT process and ovarian cancer development (Koutsaki et al., 2017[59]). As a tumor-suppressive lncRNA, SNHG10 expression is decreased in ovarian cancer tissues, and its low expression is associated with inferior overall survival and progression-free survival of affected patients. Through the SNHG10/ miR-200a-3p axis, the ectopic expression of SNHG10 or miR-200a-3p inhibitor downregulates the expression of SNAIL, upregulates E-cadherin, and decreases the migration and invasion of ovarian cancer cells (Lv et al., 2022[84]). Li et al. have studied the significance of the TMPO-AS1/miR-200c axis in the migration and chemoresistance of ovarian cancer cells. As an upregulated oncogenic lncRNA in ovarian cancers, silencing TMPO-AS1 or miR-200c overexpression decreases the migration and invasion of ovarian cancer cells both *in vivo* and *in vitro* (Li et al., 2020[63]). In this regard, increased expression of miR-200b or miR-200c decreases ZEB1 expression and migration in ovarian cancer cells (Sestito et al., 2020[111]). Yong et al. have reported that LIN28B stabilizes NEAT1, the upregulated lncRNA correlated with poor overall survival and progression-free survival, leading to the liberation of vimentin, SNAIL2, and ZEB1 expression from the inhibitory effect of miR-506. The knockdown of NEAT1 decreases the migration of ovarian cancer cells *in vitro* and reduces tumor growth in animal models (Yong et al., 2018[148]). AC005224.4 is another upregulated lncRNA in ovarian cancer cells that controls malignant cell migration via the AC005224.4/miR-140-3p/SNAI2 axis (Xiong et al., 2023[142]). In ovarian cancer cells, PTAR overexpression stimulates the EMT process, increases the migration and invasion of tumoral cells, and decreases E-cadherin expression via the PTAR/miR-101/ZEB1 axis; the *in vivo* results have demonstrated that PTAR knockdown substantially decreases tumor growth and increases the protein expression of E-cadherin (Liang et al., 2018[67]). CCAT1 is upregulated in ovarian cancer tissues, and its increased expression is associated with lymph node metastasis and higher tumor grades and stages. CCAT1 silencing decreases the migration and invasion of ovarian cancer cells via the CCAT1/miR-130b/ZEB1 axis. Besides, CCAT1 silencing and miR-130b ectopic expression upregulate E-cadherin protein expression (Cao et al., 2017[14]). SNHG1 is another upregulated lncRNA in ovarian cancer cells, involved in the migration and invasion of ovarian cancer cells via the SNHG1/miR‐454/ZEB1 axis (Wu et al., 2021[140]). MiR-145 is another ncRNA that substantially regulates the migration of ovarian cancer cells. Zhou et al. have shown that miR-145-5p expression is downregulated in ovarian cancer tissues, and its ectopic expression downregulates MMP-9 and MMP-2 expression, leading to decreased migration of ovarian cancer cells (Zhou et al., 2020[157]). Circ-0009910 is an upregulated circRNA in ovarian cancer tissues that is positively associated with poor overall survival, advanced clinical stage, and lymph node metastasis; circ-0009910 ectopic expression increases the migration and invasion of ovarian cancer cells via upregulating MMP-9 and MMP-2 expression in ovarian cancer. These pro-tumoral effects of circ-0009910 are partially reserved by miR-145 ectopic expression (Li et al., 2020[66]). Collectively, modulating dysregulated ceRNA networks can highly affect the migration and invasion of ovarian cancer cells. 

## ceRNA Networks in the Angiogenesis of Ovarian Cancer Cells

As a hallmark of malignancy, angiogenesis is pivotal in providing oxygen, nutrients, and tumor metastasis (Garrido et al., 2019[40]). The vascular endothelial growth factor (VEGF), platelet-derived growth factor (PDGF), and fibroblast growth factor (FGF)-mediated signaling pathways have been implicated in cancer angiogenesis (Figure 2(Fig. 2)). VEGFA-D, placental growth factor 1 (PIGF-1), and PIGF-2 can bind to vascular endothelial growth factor receptors (VEGFRs), activating the PI3K/Akt pathway and angiogenesis. PDGFs can bind to PDGFRs and activate the PI3K/Akt and MAPK signaling pathways, leading to tumor growth and angiogenesis. Upon activation of FGF-mediated FGFRs, the PI3K/Akt and MAPK pathways are stimulated, leading to angiogenesis and oncogenesis (Gavalas et al., 2013[42]). As important factors in hypoxia-mediated angiogenesis, hypoxia-induced factors (HIFs) are heterodimers consisting of 2 subunits, i.e., an oxygen-dependent α-subunit and an oxygen-independent β-subunit (Lv et al., 2017[85]). In hypoxic conditions, prolyl hydroxylase enzyme-mediated HIF-α hydroxylation is decreased, leading to the accumulation of HIF-α. Upon accumulation of HIF-α, the complex of HIF-α/Arnt with p300 and CBP binds to the hypoxia response elements in the promoters and enhancers of target genes (Krock et al., 2011[60]). In hypoxic ovarian cancer cells, HIF-1α silencing decreases VEGF expression. Besides, the supernatant from HIF-1α knockdown hypoxic ovarian cancer cells substantially decreases the angiogenesis of ovarian cancer cells (Bryant et al., 2010[11]).

Lin et al. have reported the significance of lncRNA DANCR/miR-145/VEGF-A in ovarian cancer development and angiogenesis. As an upregulated lncRNA in ovarian cancer tissues and cell lines, DANCR silencing inhibits ovarian cancer growth and angiogenesis *in vitro* and *in vivo* via liberating miR-145 expression and suppressing miR-145-mediated VEGFA expression (Lin et al., 2019[71]). In line with this, miR-145-5p ectopic expression downregulates Ki67, c-Myc, and VEGF expression in ovarian cancer cells. Also, ectopic expression of miR-145-5p decreases tumor volume, cancerous ascites, and diaphragmatic metastasis in animal models of ovarian cancer (Garrido et al., 2020[39]). In addition, loading miR-145 on a gold nanoplatform functionalized with FSH33 peptide can deliver miR-145 to ovarian cancer cells and decrease VEGF release and endothelial cell proliferation (Salas-Huenuleo et al., 2022[109]). As a downregulated miRNA in ovarian cancer tissues, miR-126 ectopic expression decreases tumor proliferation, invasion, and angiogenesis *in vitro* via targeting VEGFA and tumor growth *in vivo*. Of interest, the simultaneous ectopic expression of miR-126 with VEGFA has neutralized the tumor-suppressive effects of miR-126 on ovarian cancer cells (Liu et al., 2020[77]). As a downregulated miRNA in ovarian cancer tissues, miR-367 ectopic expression downregulates the mRNA and protein expression of VEGF in ovarian cancer cells and suppresses angiogenesis. Also, low miR-367 expression is associated with poor cumulative survival of affected patients. The low expression of miR-367 is associated with lymph node metastasis, higher tumor grades, and advanced tumor stages (Zheng et al., 2020[155]). Yuan et al. have highlighted the significance of NEAT1/miR-365/FGF9 in ovarian cancer angiogenesis. LncRNA NEAT1 is upregulated in ovarian cancer cells; NEAT1 ectopic expression or inhibiting miR-365 stimulates angiogenesis and upregulates VEGF and Ang-1 expression in ovarian cancer cells (Yuan et al., 2021[149]). Liu et al. have reported that MIR210HG is upregulated in ovarian cancer tissues compared to non-tumoral tissues, and its increased expression is associated with poor overall survival and disease-free survival of affected patients. MIR210HG knockdown increases HIF-1α degradation in a VHL-dependent manner, and its knockdown decreases the angiogenesis of ovarian cancer and downregulates VEGF expression (Liu et al., 2021[78]). 

## ceRNA Networks in the Metabolic Reprogramming of Ovarian Cancer

**Figure 3:**
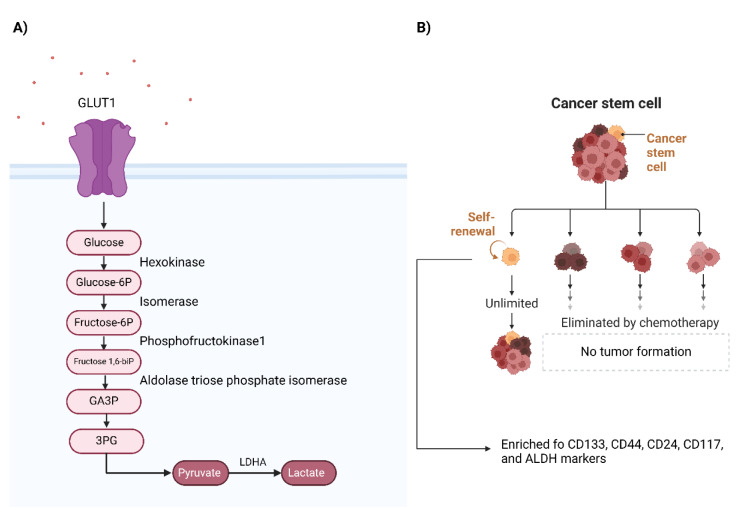
Cancer glycolysis pathway and cancer stem cell model. A) The pathway of glycolytic processes in cancer cells. B) Cancer stem cell model that demonstrates a small population of malignant cells can become resistant to anti-neoplastic treatments and can reproduce tumor bulk after treatment.

Metabolic reprogramming of ovarian cancer cells, e.g., lipid metabolism, glycolysis, and glutamine metabolism, are tumor-autonomous (Zhang et al., 2023[154]). In non-tumoral cells, ATP production is mainly dependent on oxidative phosphorylation; however, ovarian cancer cells mainly rely on glycolytic processes even in the presence of adequate oxygen supply (Ben Ali et al., 2024[9]). Ovarian cancer cells transport glucose into cells via GLUTs, Na^+^-independent sodium transporters, where hexokinase-2 phosphorylates glucose to glucose-6-phosphate. Ma et al. have reported that the inhibition of GLUT1 is associated with suppressed ovarian cancer growth both *in vitro* and *in vivo *(Ma et al., 2018[88]). In line with this, it has been reported that the expression levels of GLUT1 and hexokinase-2 are substantially upregulated in high-grade serous ovarian carcinoma compared to non-high-grade serous ovarian carcinoma, and the GLUT1 expression level is higher in advanced ovarian cancer stages compared with early ones. Also, glycolysis inhibition suppresses the proliferation of platinum-sensitive and resistant ovarian cancer cells (Xintaropoulou et al., 2018[141]). In addition, it has been shown that increased expression of hexokinase-2 is associated with tumor recurrence, chemoresistance, and poor progression-free survival in ovarian cancer patients (Suh et al., 2014[121]). After multiple steps, pyruvate kinase-1 mediates the conversion of phosphoenol pyruvate to pyruvate, which leads to the last step of glycolysis (the conversion of pyruvate to lactate) (Fadaka et al., 2017[31]) (Figure 3A(Fig. 3)). This metabolic reprogramming to increase the rate of glycolysis is also called the Warburg effect, which was discovered a century ago (Tyagi et al., 2021[128]). Nevertheless, recent findings have shown that cancer cells leverage mitochondrial oxidative phosphorylation as a backup plan; mitochondrial oxidative phosphorylation genes like ATP5MC3, ATP5ME, and ATP6AP1 are also upregulated in ovarian cancers (Wang et al., 2022[134]). The following discusses the identified ceRNA networks in ovarian cancer glycolysis as a well-studied metabolic reprogramming process. 

Lin et al. have shown that LINC00857 silencing decreases glycolysis and ovarian cancer development via the LINC00857/miR-486-5p/YAP1 axis (Lin et al., 2020[70]). LncRNA-OIP5‑AS1 is an upregulated lncRNA in ovarian cancer; lncRNA-OIP5‑AS1 knockdown downregulates hexokinase-2 protein expression and decreases glycolysis in ovarian cancer (Liu et al., 2021[80]). LncRNA-NEAT1 is another upregulated lncRNA in ovarian cancer cells and tissues, and its knockdown decreases ovarian cancer development and glycolysis via the NEAT1/miR-4500/BZW1 axis (Xu et al., 2020[143]). As a downregulated circRNA in ovarian cancer, circ-ITCH expression level is positively associated with improved overall survival of ovarian cancer patients; circ-ITCH upregulation decreases glycolysis and inhibits ovarian cancer development via the circ-ITCH/miR-106a/CDH1 axis (Lin et al., 2020[68]). CircMFN2 is an upregulated circRNA in ovarian cancer tissues and cells, and its knockdown inhibits ovarian cancer development and glycolysis via the circMFN2/miR‐198/CUL4B (Song et al., 2023[119]). 

## ceRNA Networks in the Stemness and Chemotherapy of Ovarian Cancer

Cancer stem cells are a subpopulation of malignant cells that can reproduce tumor bulk after chemotherapy via asymmetric and symmetric divisions. Cancer stem cells are resistant to the therapeutic doses of conventional chemotherapies, which can pave the way for metastasis in affected patients; therefore, identifying cancer stem cell characteristics and targeting them can improve the prognosis of affected patients (Najafzadeh et al., 2021[91]). CD133, CD44, CD24, and ALDH have been used to identify ovarian cancer stem cells (Motohara et al., 2021[90]) (Figure 3B(Fig. 3)). It has been reported that the injection of 100 CD44^+^CD117^+^ cells can reproduce original ovarian cancer; however, the injection of 10^5^ CD44^-^CD117^-^ cells does not lead to tumor recurrence (Zhang et al., 2008[151]). Silencing CD44 decreases intraperitoneal ovarian cancer growth in affected mice, decreases angiogenesis and cell proliferation, and increases apoptosis *in vivo* (Zou et al., 2014[163]). Baba et al. have shown that CD133^+ ^ovarian cancer cells are resistant to platinum-based chemotherapies and produce more aggressive cancers in animal models (Baba et al., 2009[8]). It has been shown that only 11 ALDH^+^CD133^+^ cells of human ovarian cancer cells are required to reproduce ovarian cancer in animal models, and the presence of ALDH and CD133 is correlated with poor overall survival and disease-free survival of ovarian cancer patients (Silva et al., 2011[117]). CD24^+^ ovarian cancer cells have self-renewal, quiescence, and chemotherapy resistance properties and are enriched with stemness-related genes, e.g., Nestin, Bmi-1, and Oct4. In contrast to CD24^-^ cells, 5000 CD24^+^ ovarian cancer cells are sufficient to produce ovarian cancer xenografts in nude mice (Gao et al., 2010[38]). Thus, eliminating ovarian cancer stem cells is highly important in preventing cancer relapse following anti-neoplastic treatment. The following discusses the significance of ceRNAs in stemness and chemoresistance in ovarian cancer (Table 4(Tab. 4); References in Table 4: An et al., 2017[4]; Dong et al., 2021[28]; Hou and Jiang, 2021[52]; Liu et al., 2024[76]; Wu et al., 2021[139]; Zhang et al., 2020[153]). IL21-AS1 is an upregulated lncRNA in ovarian cancer tissues and advanced-stage tumors. Silencing IL21-AS1 decreases tumor growth, increases apoptosis rate, reduces proliferation rate, and improves the survival of affected mice via the IL21-AS1/miR-561-5p/CD24 axis (Liu et al., 2024[76]). In CD133^+^ ovarian cancer cells, LINC00115 silencing decreases the expression of CD44, CD133, and NANOG. LINC00115 silencing inhibits the sphere-forming ability and improves the apoptosis rate of ovarian cancer stem cells via the LINC00115/miR-30a/SOX9 axis (Hou and Jiang, 2021[52]). LncRNA-WDFY3-AS2 is another lncRNA involved in cisplatin resistance in ovarian cancer cells. As an upregulated lncRNA in cisplatin-resistant ovarian cancer cells, WDFY3-AS2 increases tumorspheres via the WDFY3-AS2/miR-139-5p/SDC4 axis (Wu et al., 2021[139]). LncRNA-TUG1 expression level has been positively associated with higher tumor grade and FIGO stage; silencing TUG1 decreases the clonogenicity of ovarian cancer cells (Kuang et al., 2016[61]). Dai et al. have shown that lncRNA-TUG1 silencing inhibits ovarian cancer development both *in vivo* and *in vitro* via the TUG1/miR-582-3p axis (Dai et al., 2021[23]). Also, Gu et al. have reported that TUG1 is upregulated in chemoresistant ovarian cancer tissues, and its knockdown improves the chemosensitivity of ovarian cancer via the TUG1/miR-29b-3p axis (Gu et al., 2020[46]).

ZEB1 and ZEB2 have critical roles in ovarian cancer stemness and chemosensitivity (Li et al., 2021[65]; Sakata et al., 2017[108]). The knockdown of ZEB1 enhances the chemosensitivity of paclitaxel-resistant ovarian cancer cells (Sakata et al., 2017[108]). Silencing ZEB2 decreases cancer stem properties in high-grade ovarian cancer, reduces tumoral clonogenicity, and downregulates cancer stem cell-related factors, like NANOG and Oct4 (Li et al., 2021[65]). It has been shown that NEAT1 expression level is upregulated in chemoresistant ovarian cancer tissues; NEAT1 knockdown increases the chemosensitivity of ovarian cancer cells to paclitaxel both *in vivo* and *in vitro* via the NEAT1/miR-194/ZEB1 axis (An et al., 2017[4]). Regarding ZEB2, the lncRNA-HOTTIP/miR-205/ZEB2 axis has a substantial role in determining the cisplatin resistance of ovarian cancer cells. HOTTIP silencing or miR-205 upregulation considerably increases the sensitivity of ovarian cancer cells. HOTTIP knockdown decreases the stemness markers of ovarian cancer cells, e.g., NANOG, SOX2, and Oct4 (Dong et al., 2021[28]). The HOTTIP/miR-206/TBX3 axis is involved in ovarian cancer cell stemness and cisplatin resistance development. HOTTIP knockdown decreases NANOG expression and improves the chemosensitivity of ovarian cancer cells; besides, HOTTIP silencing also decreases tumor growth and the protein expression of CD44 and Ki67 in animal models (Zhang et al., 2020[153]). Tan et al. have shown that HOTTIP is an upregulated lncRNA in ovarian cancer tissues and cell lines, and its silencing decreases the clonogenicity of ovarian cancer cells via the HOTTIP/miR-148a-3p axis (Tan et al., 2021[123]). Liu et al. have reported that the pro-tumoral effect of HOTTIP ectopic expression on the clonogenicity of ovarian cancer cells is mediated via the MEK/ERK pathway (Liu et al., 2020[75]). Overall, modulating the dysregulated ceRNA networks can be a promising therapeutic option for eliminating cancer stem cells and improving the chemosensitivity of ovarian cancer cells. 

## ceRNA Networks in the Tumor Immunity of Ovarian Cancer

The immunosuppressive tumor microenvironment is one of the main culprits in protecting malignant cells from anti-tumoral immune responses, facilitating tumor growth and metastasis (Colombo et al., 2023[21]). Inhibitory immune checkpoints maintain physiological peripheral tolerance against cells; however, their pathological expression in the tumor microenvironment contributes to the immunosuppressive tumor (Pardoll, 2012[98]). The inhibitory immune checkpoint axes can be established between the cells in the tumor microenvironment, e.g., tumor-infiltrating immune cells and malignant cells (Shadbad et al., 2021[113]). CTLA-4, PD-1, PD-L1, LAG-3, TIM-3, TIGIT, and VISTA are among the inhibitory immune checkpoints that can suppress anti-tumoral immune responses (Hosseinkhani et al., 2020[51]). In this regard, delivering PD-L1-siRNA to ovarian cancer cells using PEI-FA or PEI-PEG-FA complexes makes ovarian cancer cells susceptible to T-cells (Teo et al., 2015[125]). In addition to anti-tumoral immune response suppression, tumor intrinsic PD-L1 has been implicated in cell proliferation of ovarian cancer (Clark et al., 2016[20]). In cisplatin-resistant ovarian cancer cells, tumor-intrinsic PD-L1 knockdown improves chemosensitivity, stimulates apoptosis, and arrests the cell cycle (Zuo et al., 2020[164]). Therefore, tumor-intrinsic inhibitory immune checkpoints can be important for targeted therapies. Although studies on the ceRNA networks regulating tumor-intrinsic inhibitory immune checkpoints in ovarian cancer are not extensively studied, the following discusses the currently available evidence on this topic. 

**Figure 4:**
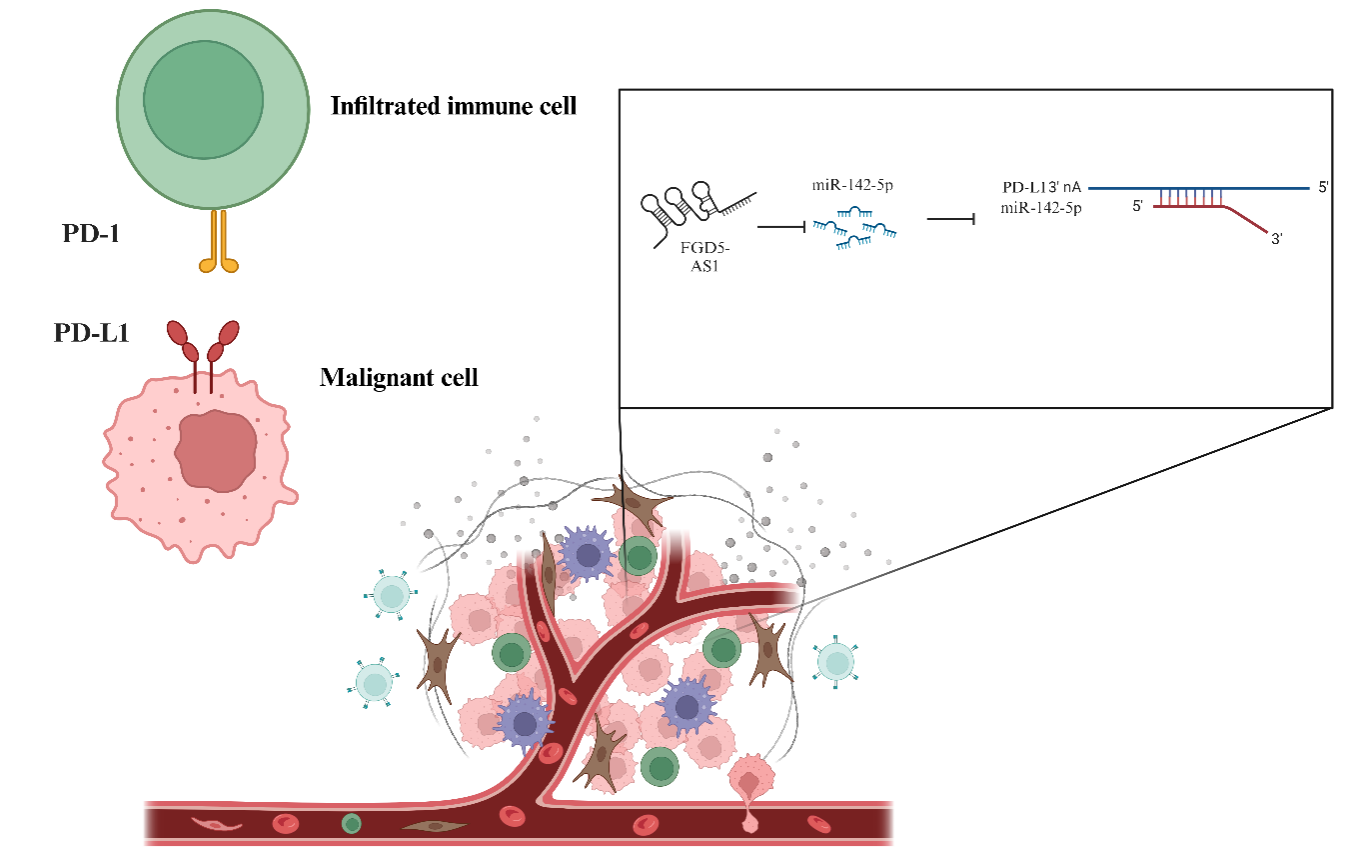
Tumor-suppressive tumor microenvironment and competing endogenous RNA in ovarian cancer. The FGD5-AS1/miR-142-5p/PD-L1 axis can promote the inhibitory immune checkpoint axis of PD-1 and PD-L1, leading to tumor growth.

Also, Sheng et al. have shown a significant negative association between PD-L1 and miR-145 in ovarian cancer tissues and miR-145 can inhibit PD-L1 expression in ovarian cancer (Sheng et al., 2020[115]). Hua et al. have shown that miR-145 is downregulated in ovarian cancer tissues, and its ectopic expression leads to the suppressed migration and proliferation of ovarian cancer cells (Hua et al., 2019[53]). Aichen et al. studied the FGD5-AS1/miR-142-5p/PD-L1 axis in ovarian cancer development. As an upregulated lncRNA in ovarian cancer tissues and cell lines, FGD5-AS1 upregulation is positively associated with lymph node positivity and higher T stage in ovarian cancer patients. LncRNA-FGD5-AS1 ectopic expression increases the proliferation, migration, and invasion of ovarian cancer cells (Figure 4(Fig. 4)) (Aichen et al., 2021[2]). Qian et al. have demonstrated that M2 macrophage-secreted IL-6 upregulates the expression of miR-21 and PD-L1 in ovarian cancer cells; also, ovarian cancer cell-secreted miR-21-containing extracellular vesicles increase IL-6 expression in M2 macrophages. In this regard, SNHG12 increases IL-6R expression in ovarian cancer cells, resulting in inhibited T-cell proliferation (Qian et al., 2020[102]). 

## ceRNA Networks in Regulating Cancer Epigenetic

Lysine methyltransferases (KMTs), lysine demethylases (KDMs), and protein arginine methyltransferases (PRMTs) are histone methylation modifiers that regulate gene expression (Chen et al., 2020[17]). KDM5A, KDM5B, KDM5C, and KDM5D are the members of the KDM5 family; the KDM5 family is one of the Jumonji C domain-containing KDM families that remove di- and tri-methyl groups from lysine 4 of histone H3 (H3K4). In this regard, H3K4me3 is present at the promoters of active genes and KDM5 proteins have been associated with transcriptional repression (Ohguchi and Ohguchi, 2022[95]). EZH2 is a histone methyltransferase that catalyzes tri-methylation of histone H3 at Lys 27. After H3K27 trimethylation, PRC1 binds to monoubiquitinated histone H2A at lysine 119 and H3K27me3, leading to chromatin compaction and transcriptional repression (Gan et al., 2018[37]). The following discusses the crosstalk between ceRNAs with KDM5 family members and EZH2 in ovarian cancer development.

Gu et al. identified the LBX2-AS2/miR-4784/KDM5C axis in ovarian cancer development. LBX2-AS2 is an upregulated lncRNA in ovarian cancer cells and tissues, and its silencing is associated with improved apoptosis rate, inhibited proliferation, migration, and stemness, and decreased tumor growth. In this regard, KDM5C upregulation attenuates the anti-tumoral effects of LBX2-AS2 silencing in terms of the proliferation, migration, stemness, and apoptosis of ovarian cancer cells (Gu et al., 2021[45]). Huang et al. discovered the lncRNA-XIST/miR-93-5p/KMT2C in ovarian cancer. LncRNA-XIST is downregulated in ovarian cancer tissues and its knockdown is associated with increased stemness of ovarian cancer cells. Of interest, KMT2C upregulation decreases the stemness mediated by lncRNA-XIST silencing in ovarian cancer cells (Huang et al., 2020[55]). LncRNA-ATB is an upregulated lncRNA in ovarian cancer tissues and its expression is positively associated with poor survival of patients. It has been shown that lncRNA-ATB inhibition decreases the enrichment of EZH2 in the CDX1, LATS2, FOXC1, and E-cadherin promoters and decreases the histone trimethylation of their promoters in ovarian cancer cells (Chen and An, 2021[16]). Silencing lncRNA-HOTAIR increases apoptosis and decreases the migration, invasion, and spheroid-forming ability of ovarian cancer cells; Dai et al. have suggested that EZH2-mediated methylation is one of the mechanisms of lncRNA-HOTAIR pro-tumoral effect (Dai et al., 2021[24]). 

## ceRNA Networks as Biomarkers for Ovarian Cancer Patients

**Figure 5:**
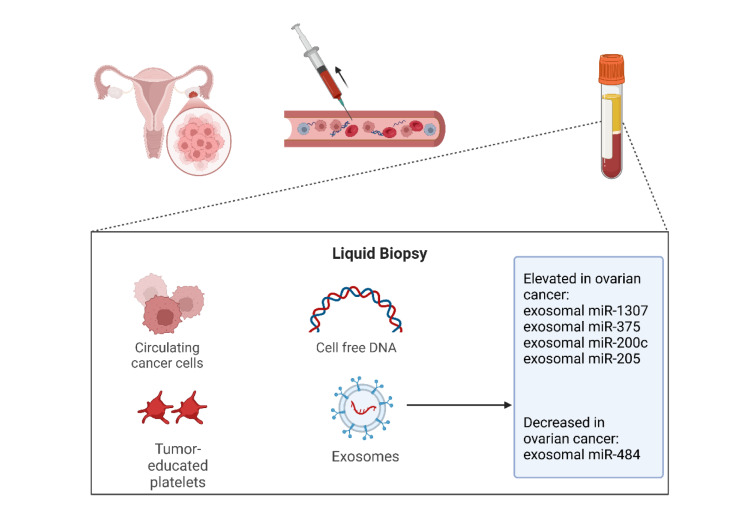
Liquid biopsy for ovarian cancer. Tumor-released factors, including circulating tumor cells, tumor-educated platelets, circulating tumor DNA, and exosomes, can be leveraged as biomarkers for ovarian cancer.

Due to the lack of appropriate screening approaches and related symptoms, ovarian cancer is usually diagnosed in its late clinical presentation (Asante et al., 2020[6]). Despite the clinical application of the serological study of CA-125 in ovarian cancer patients, this approach has limited clinical efficacy in terms of ovarian cancer diagnosis. A randomized clinical trial with a median follow-up of 15 years has indicated that transvaginal ultrasound and CA-125 levels have no mortality benefit (Pinsky et al., 2016[101]). Liquid biopsy is a minimally invasive, feasible approach that provides ample opportunity for dynamic monitoring of the malignancy and the diagnosis and prognosis of affected patients. Over the last decade, liquid biopsy has attracted the attention of many researchers to facilitate the diagnosis, treatment response, and prognosis of patients (Paracchini et al., 2021[97]). The concept of liquid biopsy is based on investigating the tumor-released factor in human fluids, like circulating tumor cells, tumor-educated platelets, circulating tumor DNA, exosomes, and cell-free RNA (Zhu et al., 2022[158]). The following provides an overview of the recent evidence on the significance of circulating ceRNA components in the diagnosis and prognosis of ovarian cancer patients (Figure 5(Fig. 5)).

The serum-derived exosomal miR-1307 and miR-375 are upregulated in ovarian cancer patients. The combination of serum-derived exosomal miR-1307 and miR-375 with CA-125 level offers an AUC of 0.977 for ovarian cancer diagnosis. Also, the serum exosomal miR-1307 is elevated in the higher stage compared to early-stage ovarian cancer (Su et al., 2019[120]). The serum-derived exosomal miR-200c is upregulated in high-grade serous ovarian carcinoma patients compared to patients with benign ovarian cysts, borderline ovarian tumors, and non-high-grade serous ovarian carcinoma patients. In addition, the combination of miR-145 and miR-200c with the CA-125 level leads to a sensitivity of 100 % and a specificity of 55 % for ovarian cancer diagnosis (Kim et al., 2019[58]). Chen et al. have shown that the serum level of miR-125b is downregulated in epithelial ovarian cancer patients, and patients with advanced tumor stage and lymph node metastasis have lower levels of miR-125b. Besides having prognostic significance, the combination of miR-125b serum level with CA-125 markedly increases the diagnostic power of serum CA-125 levels in epithelial ovarian cancer patients (Chen et al., 2020[19]). Zhu et al. have reported that miR-205 exosomal level is increased in ovarian cancer patients, and combining miR-205 exosomal level with CA-125 and protein HE4 increases the sensitivity and specificity of ovarian cancer diagnosis to 100 % and 86.1 %, respectively (Zhu et al., 2022[162]). Talaat et al. have shown that miR-21 expression is upregulated in the serum of ovarian cancer patients, and its sensitivity and specificity are 96 % and 88 %, respectively (Talaat et al., 2022[122]). In this regard, the plasmatic levels of miR-21 and miR-26b are upregulated and downregulated in ovarian cancer patients, respectively. Besides the prognostic values of miR-21 and miR-26b expression, their combination levels have a sensitivity and specificity of 87.6 % and 90.4 %, respectively (Song et al., 2020[118]). Ge et al. have reported that the combination of the plasmatic levels of has-circ-0007288 and has-circ-0003972 with CA-125 yields a higher diagnostic value compared with the CA-125 level for ovarian cancer diagnosis (Ge et al., 2022[43]). Gahlawat et al. identified a signature of seven plasmatic miRNAs, i.e., miR-486, miR-92a, miR-320b, miR-200c, miR-335, miR-320c, and miR-375, that has an AUC of 0.87 for ovarian cancer diagnosis, and the combination of this panel with CA-125 increases the diagnostic power in terms of case detection (Gahlawat et al., 2022[36]). A decrease in the level of miR-34a-5p and miR-93-5p has been associated with improved progression-free survival and overall survival of ovarian cancer patients treated with perioperative chemotherapy and interval debulking surgery, respectively (Robelin et al., 2020[106]). Besides the diagnostic significance of plasmatic miR-484 and improving the diagnostic power of CA-125, low levels of miR-484 are associated with poor overall survival and progression-free survival in ovarian cancer patients (Zhang et al., 2020[152]). The recent advances in next-generation sequencing and computational biology approaches have enabled us to study big data on various human conditions, like ovarian cancer (Nomiri et al., 2022[93]). Therefore, this research area can introduce novel biomarkers for the diagnosis and prognosis of ovarian cancer patients that can be used in clinical practice. 

## Tumor Heterogeneity and ncRNAs

Ovarian cancer is a heterogeneous disease; malignant cells are classified into various subpopulations based on their expression patterns (Winterhoff et al., 2017[137]). The vast intra-tumor, inter-tumor, and temporal heterogeneity of malignant cells have been implicated in why patients respond differently to the standard treatment and develop metastasis following treatment (Abdoli Shadbad et al., 2021[1]). In this regard, studying the expression profile of cells residing in the tumor microenvironment of patients can reveal which tumor suppressors and oncogenes are dysregulated; thus, these data aid in tailoring personalized, targeted therapies for affected patients (Abdoli Shadbad et al., 2021[1], Shadbad et al., 2021[114]). In addition, the tumor tissues also contain normal cells; therefore, single-cell RNA analysis can provide more in-depth insights compared to bulk tumor analysis (Alessio et al., 2020[3]). Recent advances in single-cell ncRNA sequencing approaches have enabled us to study the ncRNA expression profile of cells during their development (Fan et al., 2015[32], Hücker et al., 2021[56]). In this regard, Pang et al. have leveraged single-cell RNA sequencing techniques to depict the transcription factors and lncRNA implicated in acquiring the invasion phenotype from cancer stem cells in glioblastoma (Pang et al., 2019[96]). Besides, the expression profile of lincRNA, as a ncRNA, is more specifically expressed compared to protein-coding RNAs (Wang and Roy, 2017[130]). Overall, applying single-cell sequencing data can be integral in studying the biological aspect of malignant cell evolution and tailoring personalized, targeted therapies.

## Concluding Remarks

The dysregulated ceRNA networks are highly implicated in cancer development, progression, and metastasis via upregulating oncogenes and downregulating tumor-suppressive genes in ovarian cancer. Over the past decades, accumulating evidence has shed light on the miRNA/mRNA, circRNA/ miRNA/mRNA, and lncRNA/miRNA/ mRNA axes in the various aspects of ovarian cancer development. Since these ncRNAs can directly or indirectly suppress the gene expression and signaling pathways implicated in cancer hallmarks, they are considered valuable targets for treating various solid cancers, like ovarian cancer. Regulating the lncRNA- and circRNA-mediated ceRNA networks can inhibit oncogenesis and increase cancer immunotherapy and chemotherapy responses. In addition to their therapeutic potential, circulating ncRNA can serve as promising biomarkers for the diagnosis and prognosis of ovarian cancer patients; this research area has become more interesting with the advances in computational biology and the promising potentiality of liquid biopsy. For this aim, combining* in silico* studies with *ex vivo*, *in vitro*, and *in vivo* ones improves our understanding of these axes in ovarian cancer development. Meanwhile, the revolutionary advances in single-cell sequencing techniques can aid us in studying the trajectory of malignant cell evolution and the impact of ncRNA and ceRNA on its fate, as well as tailoring personalized anti-neoplastic treatments. Although the current study comprehensively reviewed the identified axes in each context of ovarian cancer hallmarks, one ceRNA axis can regulate multiple ovarian cancer hallmarks. Given the promising potential of modulating dysregulated ceRNA networks for ovarian cancer diagnosis, prognosis, and treatment, further studies are needed to apply single-cell RNA sequencing approaches in the context of ovarian cancer. 

## Declaration

### Ethics approval and consent to participate

Not applicable.

### Consent for publication

Not applicable.

### Availability of data and materials

Not applicable.

### Competing interests

The authors declare that they have no competing interests.

### Funding

None.

### Authors' contributions

RD: Conceptualization, investigation, writing - original draft, and writing - review & editing. FNO: Writing - review & editing, conceptualization, and visualization, and MAS: Conceptualization, writing - review & editing, supervision. All authors read and approved the final manuscript.

### Acknowledgments

We appreciate all the researchers working on ncRNAs and ceRNA networks in malignancies. The figures are prepared using BioRender. 

## Figures and Tables

**Table 1:**
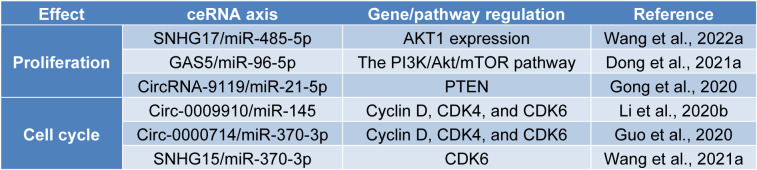
ceRNA networks in the proliferation and cell cycle of ovarian cancer

**Table 2:**
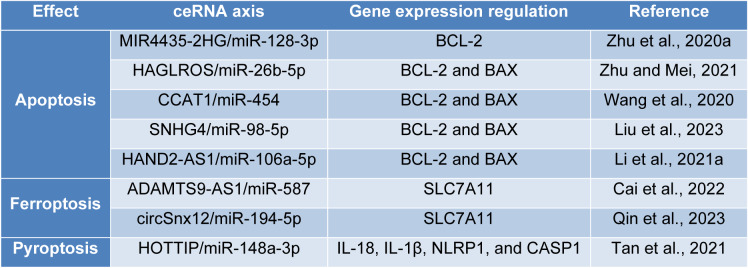
ceRNA networks in the apoptosis, ferroptosis, and pyroptosis of ovarian cancer

**Table 3:**
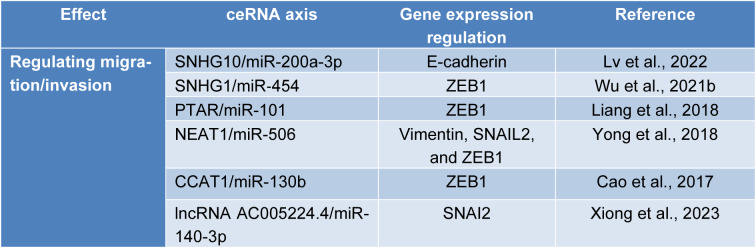
ceRNA networks in the migration and invasion of ovarian cancer

**Table 4:**
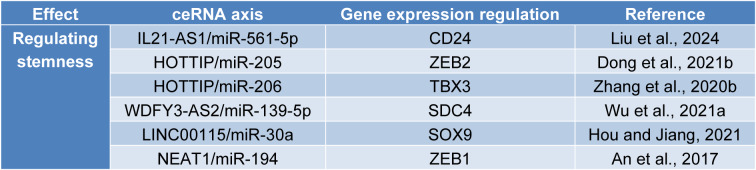
ceRNA networks in the stemness of ovarian cancer
